# A Systematic Review of Children’s Physical Activity Patterns: Concept, Operational Definitions, Instruments, Statistical Analyses, and Health Implications

**DOI:** 10.3390/ijerph17165837

**Published:** 2020-08-12

**Authors:** Thayse Natacha Gomes, Peter T. Katzmarzyk, Sara Pereira, Mabliny Thuany, Martyn Standage, José Maia

**Affiliations:** 1 Department of Physical Education, Federal University of Sergipe, São Cristóvão-SE 49100-000, Brazil; mablinysantos@gmail.com; 2 Pennington Biomedical Research Center, Baton Rouge, LA 70808, USA; peter.katzmarzyk@pbrc.edu; 3 CIFI2D (Centre of Research, Education, Innovation and Intervention in Sport), Faculty of Sport, University of Porto, 4200-450 Porto, Portugal; sara.s.p@hotmail.com (S.P.); jmaia@fade.up.pt (J.M.); 4 Department for Health, University of Bath, Bath BA2 7AY, UK; spsms@bath.ac.uk

**Keywords:** physical activity pattern, children, definition, measurement, health

## Abstract

Despite the widespread use of the expression “physical activity pattern” (PAP), there apparently is no general consensus regarding its definition. This systematic review aimed to examine available research focussing on (1) definitions of PAP, (2) instruments/techniques used to describe PAP, (3) statistical approaches used to analyse PAP, and (4) implications of PAP on children’s health. A systematic review of the available literature was done to identify studies published up to October 2019, and 76 studies were eligible. None of the studies presented a formal definition of PAP; a wide range of instruments were used to investigate children’s PAP, and most of the revised studies did not explicitly present a formal statistical model to define PAP. Twenty-four papers purported to examine associations between PAP and health indicators. The review highlights no consensus on a clear PAP definition whatever the instrument used to capture it, and we did not find any agreement regarding how best to analyse PAP. We suggest that PAP should be used when targeting the investigation of similarities/dissimilarities, as well as stabilities and/or changes in children’s PA at an intra-personal level. In sum, PAP should be used to best describe individual streams of behaviours, and not exclusively PA levels/intensities.

## 1. Introduction

Physical activity (PA) is positively associated with numerous physical, psychological, and cognitive health benefits in children and youth [1]. Despite the development of science-based global guidelines for achieving these health benefits [2], epidemiological data suggest that PA among youth is declining in many countries, and that pronounced proportions of children do not meet the recommendations [3]. A large number of studies have investigated putative correlates of children’s PA, focussing mainly on understanding the differences in mean levels of PA at various intensities (i.e., sedentary, or light, moderate, vigorous) [4]. Yet, children of the same age and sex differ widely in the way they engage in the manifold expressions of their PA [5]. Indeed, not only does every child’s PA differ in duration and intensity, but it also varies according to spaces/places [6], segments of the day [7], weather conditions [8], and seasons [9]. Such complexity has raised the bar and challenged researchers to also understand children’s PA patterns (PAP) so as to better grasp its meaning and relevance as well as health benefits [10,11].

Surprisingly, there is no consensus on a definition of PAP, which has led to using different procedures to capture and describe its expressions in children. For example, Laguna et al. [12] defined PAP as moderate-to-vigorous physical activity (MVPA) performed from 07:00 to 00:00, taking into account the weekly mean, as well as means for weekdays and weekend days. Ruiz et al. [7] operationalised PAP as the duration of MVPA, as well as the intervals between MVPA episodes alongside the day. Alternatively, Muntanr-Mas et al. [11] used a questionnaire to investigate children’s PAP that entailed not only the frequency of involvement in PA or physical inactivity (hours∙week^−1^), but also several factors related to the context within which the PA was performed. PAP have also been investigated with respect to intensity as well as associations with daily weather conditions [8].

In addition to definitional differences, there has also been substantial variation in the methods and statistical approaches to capturing and analysing PAP. Studies differ not only in design (cross-sectional, longitudinal, intervention), but also in terms of the instruments used to estimate PA, varying from subjective means (self-report questionnaires, self-report diaries/logs, direct observation) to devices (accelerometers, pedometers, heart rate monitors). Similarly, researchers have used differing analytical approaches to understand variations in PAP, including analyses such as t-test [11,12], chi-square [11], ANOVA [13], cluster analysis [14], factor analysis [15], and multilevel models [8]. Such disparities in designs, methods, and statistical procedures may reflect the absence of a clear-cut definition of PAP.

Simply put, a pattern is something that repeats itself. Yet, the ways in which such repetitions are described, measured, analysed, and comprehended involve different classical methods [16,17] as well as data-mining approaches [18,19,20] which are still open to debate [21]. In children’s lives, their PAP is also governed by their family and school contexts. For example, among Pima Indian children, Johnson et al. [22] reported that PAP on school days differed between the communities studied (i.e., Pima children living in Pima communities versus their public school counterparts). Such findings suggest that certain school characteristics, namely more areas for children to play before and after school and also during school recess, may be associated with children’s PAP. Additionally, and although the participants in the study were not involved [22], the school in the Pima community participated in health intervention programs years earlier. Therefore, it is plausible that some teachers still employed strategies learned during the intervention program related to increasing PA (e.g., provision of class walking and activity breaks).

Rowlands and Hughes [13] investigated differences in PAP in children across seasons (summer and winter) and across school and vacation time (within those seasons). Results of their work showed differences across seasons, between week and weekend days, and a significant season-by-school interaction also emerged. Specifically, they reported that children tend to be more physically active during summer than winter, and during the week than weekend days. Moreover, their data also showed that children engage in increased activity during summer vacations compared to summer school, winter vacation, and winter school periods. The authors highlighted the relevance of this information, given that the role of season and school time on children’s PAP may influence the identification of the relationship between PA and health in youth.

To the best of our understanding, there is no formal definition of PAP, i.e., a concise definition that may help explore children’s daily or weekly serial data obtained from accelerometry or a similar device. Yet, the absence of such a definition has not hindered researchers from their quest of trying to describe and understand children’s PAP. As suggested by Berman et al. [23], understanding the differences in spontaneous involvement in PA among children, with their different PAPs, could yield relevant findings to better understand its impact on health/disease and quality of life, leading to the development of new intervention approaches. However, without a consensus regarding the complexities and meaning of PAP, this effort seems hard to systematically develop. Accordingly, the aims of the present study were to review available research focussing on the following: (1) definitions of PAP; (2) instruments and techniques used to describe PAP; (3) statistical procedures used to analyse PAP; and (4) implications of PAP for children’s health.

## 2. Materials and Methods

### 2.1. Protocol and Registration

This systematic review was registered in the International Prospective Register of Systematic Reviews (PROSPERO; registration number CRD42018096728), and was performed following the PRISMA (Preferred Reporting Items for Systematic Reviews and Meta-Analyses) guidelines [24].

### 2.2. Search Strategy

A systematic review of the literature was performed using four electronic bibliographic databases (PubMed, Scopus, Academic Search Complete, and SPORTSDiscus) using the following search terms, “physical activity pattern*” and “child*”. Bibliographic records for the identified papers were extracted into EndNote reference manager software (version X8, Thomson ResearchSoft^®^), where duplicated results were identified and removed. Titles and abstracts of potentially relevant papers were screened, and those selected were screened independently in full text by two reviewers. To be included in the present review, eligible papers were confirmed by the two reviewers, and if discrepancies arose, they were solved by discussion between the reviewers.

### 2.3. Eligibility Criteria

Original peer-reviewed articles published from the year of database inception to October 6, 2019 were eligible for inclusion. Additional eligibility criteria included: (1) published in English, (2) the purpose was stated as investigating children’s PAP and presented as a study aim or clearly detailed in the Methods section; (3) the sample was comprised of children aged 6–11 years or with mean age between 6.0 and 11.49 years, or in the case of samples with older/younger children, the results should be presented by age or most of the sample should be included in this age range (information clearly presented in the studies); (4) the sample should not be only comprised by children with special needs; yet, if the sample comprised children with special needs and also children with typical development, the study was included.

### 2.4. Methodological Quality Assessment and Network

Then, the articles selected were analysed for their data quality, taking into account seven quality criteria developed *ad hoc* that were adapted for previous studies [25,26] (Table 1), which were scored on a three-point scale, and the sum of these points (from 0 to 14 points), meaning the methodological quality rating (which was represented in a percentage scale). This procedure was performed by two independent reviewers, separately, and if discrepancies arose, they were solved by common agreement. A bibliometric analysis was performed by a network created using the software Gephi, aiming to show the networks defined by the authors of the revised papers (Supplementary file Figure S1).

## 3. Results

**Figure 1:**
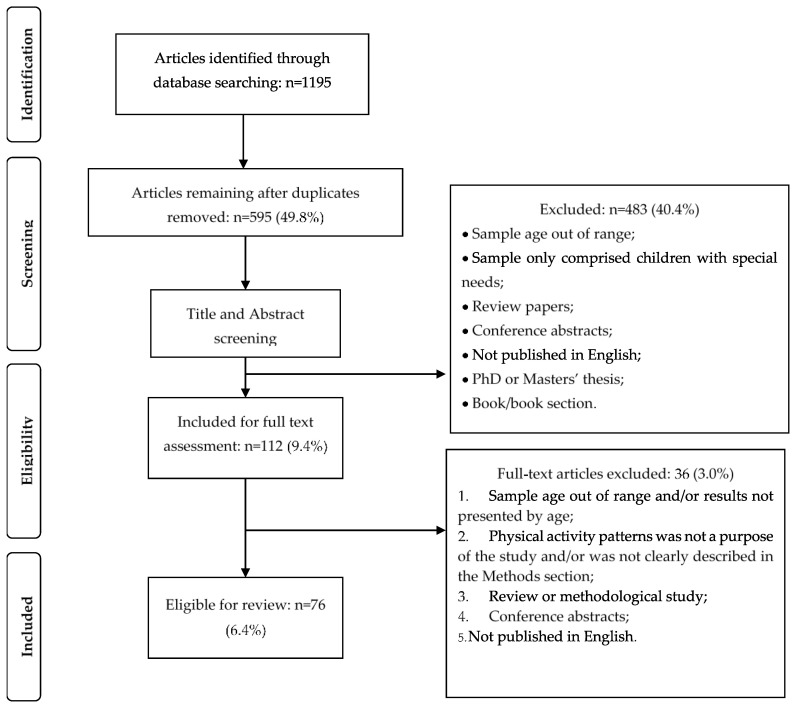
PRISMA flow diagram of screening process.

Figure 1 shows the flow diagram of the article identification process. Using the aforementioned keywords, the electronic database search produced an initial total of 1195 results. After excluding duplicates, 595 were screened by their titles and abstracts. This process allowed a further exclusion of 483 papers, resulting in 112 papers for full textual assessment. Finally, 76 studies were found to be eligible for this review.

From the included studies, relevant data were extracted, as per our aims: sample characteristics [size, age (mean and/or range), country], methods used to measure PA and to describe PAP, as well as possible links with health risk factors. Most of the studies presented within the retrieved papers, as expected, were conducted in Europe and/or North America, with a few being carried out in Asia, Africa, Oceania, and South America. Study samples ranged from 15 [23] to 17,500 [27] subjects, and with the exception of one study, all investigated both boys and girls.

### 3.1. Methodological Quality

Results regarding the papers’ quality rating revealed that the highest mean score was observed for studies that used mixed instruments to determine/measure PAP (9.45 points, meaning a quality score percentage of 67.53%), followed by those that used pedometers (9.15 points, percentage of 65.31%), accelerometers (9 points, with a quality score percentage of 64.29%), heart rate monitors (8.67 points, 61.90%), and questionnaire/observation methods (8.18 points, percentage of 58.40%). All of the articles were scored as 0 for Q3, and the most of them received a score of 1 for Qs 4, 5, 6, and 7.

### 3.2. Physical Activity Pattern Definition

There is no consensus regarding a definition of PAP in conceptual and/or operational terms. As far as we could tell, no paper included in this review presented a formal definition. This implies that different schemes to tackle PAP were used, which varied according to the method used to measure PA. Consequently, most studies approached PAP considering children’s involvement in different PA intensities (sedentary, light, moderate, vigorous, very vigorous), which were obtained according to specific accelerometer or pedometer cut-points or even based on physiological measures such as heart rate. Further, some studies also presented PAP as the type of activity children performed, as well as their sports participation.

In some cases, segments of the day as well as different days of the week, or even different seasons, were used to define PAP. As such, some researchers focused their attention on describing children’s PA in different segments of the day, i.e., during school (all school time, breaks, physical education classes), before/after school, and in the evening, trying to find differences among such time segments. In contrast, others were interested in describing children’s PA during week/school days and during the weekend, contrasting these days with the aim of understanding if the child’s PA varied according to the days of the week. Further, others were interested in analysing children’s PA in different seasons, usually fall/winter season and spring/non-winter season.

### 3.3. Instruments Used to Determine PAP

A wide range of instruments were used to determine PAP including accelerometers [8,9,12,28,29,30,31,32,33,34,35,36,37,38,39,40,41,42,43,44,45,46,47,48,49,50,51,52,53,54,55,56], pedometers [22,57,58,59,60,61,62], heart rate monitors [63,64,65,66,67,68,69,70,71], questionnaires/diaries [10,11,27,72,73,74,75,76,77,78,79,80,81,82,83], and observational methods [23,84]. A few studies combined instruments, such as accelerometers and GPS [6], accelerometers and questionnaires or diaries [14,85,86,87,88], accelerometer and heart rate monitors [89], pedometers and questionnaires [90,91], or heart rate monitors and questionnaires [92,93] (Table 2, Table 3, Table 4, Table 5 and Table 6).

#### 3.3.1. Accelerometry Data

Thirty-nine studies used accelerometers to assess PAP, from which 32 relied solely on accelerometers, while 7 studies employed them in combination with another instrument (Table 2). In general, children were monitored from 3 days to 2 weeks consecutively (in seven of the studies, children were monitored more than once [8,9,29,52,53,54,55]), and most of the data were collected during children’s awake period only; only in 4 studies [32,34,50,56] did children wear the accelerometer for 24 h/day.

Total PA or its different levels/intensities were computed in all the studies aiming to express children’s PAP. In other words, researchers were primarily focussed on describing/identifying the amount of time (or the percentage of time) children spent in PA of different intensities (including sedentary behaviour). The main idea was to determine how these activities vary across the day (considering different segments of the day) [14,28,29,35,39,41,42,44,46,47,53,56,85,86,89], across week and weekend days [12,14,29,30,33,39,41,43,44,45,47,48,50,55,56,85,86], or even how children respond to an intervention program [52,54].

When PAP was described across the day, the following approaches were mainly used: (1) describing PA per hour or the average of the measured days (at different intensities); (2) comparing PA levels taking into account different segments, namely time spent at school (during classes, physical education classes, breaks) and time out of school (before and after school, transportation mode to/from school; activities done during leisure time, activities at home, parks, sports participation); (3) describing the amount of time children spent in MVPA, and even the percentage of children complying with MVPA guidelines; (4) time spent in sedentary behaviours. Further, there was a systematic use of mean/average time in PA from the valid/measured days alongside the studies, with few studies focusing on describing PAP daily. However, when this approach was used, authors focused their efforts on children’s MVPA (the time spent in each day, as well as the number of days they comply with the PA guidelines) [12,35,41,42,44]. In addition, a large number of papers also focused on comparing PAP between weekdays (or school days) and weekend days, considering the average value of the days (Monday to Friday for weekdays; Saturday and/or Sunday for weekend days) [8,12,14,29,30,33,39,41,43,44,45,47,48,50,55,85,86], and two studies investigated the effect of an intervention program on children’s PAP comparing differences in mean minutes children spent in different PA intensities and sedentariness/rest [52,54]. None of the studies investigated children’s PAP by day and/or by hour at the same time (when authors focussed in daily PAP, the daily averages were used; while when the focus was on hourly PAP, averages of each intensity of PA per each hour were used).

The use of questionnaires, in association with accelerometers, allowed some authors to identify the travel mode children used to/from school/home [85,86], the type of activities usually engaged in or the activities they performed while the accelerometer was used [87], as well the estimation of the energy expenditure, based on activities recorded in the diary [88], and also the time spent in sedentary behaviours as well as the kind of sedentary activities typically engaged in [14]. Additionally, the combined use of GPS with an accelerometer provided information regarding the places and contexts that children tend to engage in PA, allowing these to be associated with PA intensities [6]. Furthermore, when accelerometers were used in combination with heart rate monitors [89], information from this last instrument was used as cut-point to classify children’s PA levels considering the maximum age-related heart rate.

#### 3.3.2. Pedometry Data

Pedometers were used in only 9 studies (seven [22,57,58,59,60,61,62] of them used only the pedometer, and two [90,91] employed pedometers in combination with a questionnaire) (Table 3). The pedometers were worn between four and seven days, only during awake periods, and in one of the studies, children were monitored across one academic semester (i.e., by using the pedometer for 7 days per month).

Similar to the accelerometer studies, authors were mainly focussed on describing PAP according to the number of steps children achieved in a typical day, using the average number of steps [22,57,58,59,60,61,62,90,91] or classifying children according to some daily recommendation [61,90]. One study focussed only on school time, meaning that the pedometer was just used during the school period (from 8:00 to 15:00) [22].

Step counts were presented by differences along a typical day comparing different daily segments (in general, segments “in school” and “out of school”) [57,58,59,62,90], comparing week and weekend days [58,59,60,61], and also analysing differences across seasons (fall, winter, and spring) [58,91].

Given that there is no universal step cut-point to classify children into different PA levels, few studies used this approach. In fact, one study classified children as low or high active, taking into account the median of their daily steps as the cut-point [90], while another used an existing cut-point recommendation [61]. The combined use of questionnaires provided authors information regarding children’s travel mode [90] as well as time (hours/day) spent in different physical activities and sedentary behaviour [91].

#### 3.3.3. Heart Rate Monitoring

Heart rate monitors were used in 11 studies [63,64,65,66,67,68,69,70,71,92,93], but only two used them concomitantly with a questionnaire [92,93] (Table 4). The timeframe for heart rate monitoring ranged from 3 h to 4 days. Only one study monitored children’s heart rate 24 h/day [69], and in another study, children were also monitored during the weekend (two weekdays and one weekend day) [93]. Differences in PAP between seasons were also investigated in one of the papers (autumn and summer) [63].

Heart rate was also used to classify children according to their PA levels, although cut-points varied across the studies. Generally, authors reported the time, or percentage of time, children spent in different intensity activities (low, medium, high, or MVPA) across the monitored time, and the PAP was described hourly (when children used the monitor during one day [71,92] or the average value was used [63,64,65,66,67,68,69,70,71,92,93]. In addition, and when used in combination, questionnaires provided additional information regarding the type, time, period, and place of activity [92,93].

#### 3.3.4. Questionnaires

The use of questionnaires to study PA has a long history in epidemiological studies with large samples given that cost and administration burden are low. We identified 15 studies that used questionnaires to study children’s PAP [10,11,27,72,73,74,75,76,77,78,79,80,81,82,83], with information being reported by children or by their parents (Table 5).

The information derived from questionnaires was related, in general, to children’s PA engagement on typical days. Often, participants were asked to report time spent in activities of different intensities (light/low, moderate, vigorous, and sedentary) in the previous month [77], or in a typical week [11,27,74,75,79,80], or in the last week [74,76,78], or in a typical day [10,72,73,81], or even in periods out of school (leisure time), as well as their current PA level [78]. Furthermore, in some studies children were also asked to report the estimated time spent in screen entertainment or other sedentary behaviours [10,11,72,73,74,75,76,78,81]. When parents were responsible for answering the questionnaire, their information was related to the time (minutes/hours), and/or frequency (days) with which their children were involved in PA of different intensities. In some reports, the information also allowed the estimation of MVPA, which was used as time (minutes/day [10,11,27,75,76,77], or minutes/week [80]) or to classify children as having complied, or not, with the MVPA guidelines [11,72,77], and also by an estimation of metabolic equivalent [81], or even the use of a score derived from the questionnaire [83].

Given the substantial range of questionnaires used to assess PA and that the information that they provide varies substantially, it is not easy to cluster the different strategies that authors have used to determine PAP from questionnaires. However, independent of the output variable used, selected papers usually focussed on expressing PAP according to time or frequency spent in PA, compliance or not with MVPA guidelines, average differences in PA and sedentariness among different segments of the day (school segments and out of school), or between week and weekend days, travel modes, or even the child’s participation in sports.

#### 3.3.5. Observation

Only two studies used an observational approach to study children’s PAP (Table 5). To describe PAP, Berman et al. [23] followed an observational protocol of 12 h/day divided into four-hour time blocks (8:00–12:00; 12:00–16:00; and 16:00–20:00), which were further divided into consecutive 30 min time blocks. For each one of these blocks, children’s PA categories and intensity were coded, and an estimation of VO_2_ (mL/min/kg) computed. Then, bouts of different intensities of PA were categorised as being “high” or “low” based on whether the estimated VO_2_ was above or below anaerobic or lactate threshold during the observational period. Within the context of school physical education classes, Corbin and Pletcher [84] described PAP according to children’s involvement in unorganised, low organised, and organised play situations during classes. An activity index was derived that allowed for an estimation of energy expenditure, as well as the percentage of time that children spent in different activities during physical education (sitting, walking, etc.). This approach was also used to determine the percent of time that children were actually active.

### 3.4. Statistical Procedures

Most of the reviewed studies did not explicitly describe the use of a formal method to determine children’s PAP. From the few studies where this information was presented, the methods included profile analysis [71,92], cluster analysis [14,23], spectral analysis [23], principal component analysis [74], and ratios [40].

Profile analysis was used to compare differences between sex [92] or between experimental and control groups [71] across pre-determined heart rate categories. Berman et al. [23] used two procedures to determine PAP (cluster and spectral analysis), where the spectral analysis was used to determine the existence of recurring patterns of activity bouts in each 24-min period, while the cluster analysis allowed the authors to identify the occurrence and duration of these bouts. Cluster analysis was also used by Jago et al. [14] to identify groups of children with similar behavioural profiles regarding their PA and sedentary behaviours.

Principal components analysis was the method chosen by Antonogeorgos et al. [74] to obtain children’s PAP, based on the inter-correlations between PA variables, where PAPs were apparently revealed. Using a different approach, Loprinzi et al. [40] described PAP according to ratios of different PA markers, namely MVPA/sedentary behaviour, light PA/sedentary behaviour, and total PA/sedentary behaviour, where ratios ≥ 1 implied that children were relatively more engaged in MVPA, light PA, or total PA than in sedentary behaviour.

### 3.5. Physical Activity Patterns and Health

From the studies included in the present review, 24 studied associations between PAP and health indicators in children, including body composition [40,82,93], nutritional status [11,12,28,34,45,50,57,59,60,66,67,70,72,73,74,84,89,90], cardiovascular [78] or metabolic risk factors [51], and bone mineral density [83].

Regarding body composition, only three studies investigated associations with PAP. Here, disparity existed between the reported results. Al-Nakeeb et al. [93] did not find significant relationships between body fat percentage and time spent in MVPA, whereas Loprinzi et al. [40] described significant differences in different body composition indicators [body mass index (BMI); waist circumference; triceps and subscapular skinfolds; android, gynoid and body fat percentage] between children who met the MVPA recommendations and engaged in more light PA than sedentary behaviour versus those who did not comply with the MVPA guidelines and engaged in less light PA than sedentary behaviour. In contrast, Bosch et al. [82] reported that actively commuting children were less likely to have high fat mass than their passive commuters peers, and those who were engaged in sports less than once a week were less prone to have high fat-free-mass when compared to daily active children.

The relationship between PAP and cardiovascular/metabolic risk markers was investigated in only two studies [51,78]. In the first, Schmidt et al. [78] used children’s self-reported PA (actual and regarding the past 14 days) and categorised children into five groups based on their actual PA (i.e., inactive; relatively inactive; light PA; moderate PA; and vigorous PA). The authors also took into account the number of days that the children were involved in PA of different intensities (hard exercise, easy exercise), multimedia usage, and their annual sports participation. Results showed significant and negative associations between PAP and markers of cardiovascular, yet there were different by sex; i.e., in boys, PA was correlated with total cholesterol and triglycerides; whereas in girls, correlations were observed for body fat percentage and BMI. In Aadland et al.’s study [51], with accelerometry information, the association of children’s PA volume and patterns with metabolic risk factors was investigated. It was concluded that there was a strong negative association between metabolic health and vigorous PA, yet this association was weak with moderate and light PA, and no association was observed with sedentariness. Further, it was reported that the association between metabolic health and PA seems to be determined by accelerometer epoch settings, whereby short epochs appeared more favourably associated with metabolic health than long epochs. Regarding the relationship between PA and bone mineral density, the only study included in the present review addressing this purpose did not find any significant association [83].

The majority of the reviewed studies focussed on the possible links between PAP and nutritional status. Here, results were shown to be divergent. Five [28,66,67,73,90] of the 18 studies did not find any significant association, meaning that children’s PAP (levels/intensity) did not differ in groups with different nutritional statuses. It seems important to note that despite most of the studies focussing their attention towards understanding this relationship in children with normal weight and those with excess weight, Benefice et al. [66,67] sampled malnourished children and compared their PAP with normal weight children’s PA and reported no differences. On the contrary, other studies reported PAP-related differences between normal weight children and their overweight/obesity peers, favouring the normal weight group (e.g., they tend to be more active or comply more with the MVPA guidelines). Further, some sex differences in this association were also observed. For example, Butte et al. [34], Sigmund et al. [89], and Williams et al. [50] reported this difference only among boys (in girls, no significant association was observed), but a slightly difference in methods/results in these studies should be highlighted. While the first two [34,89] investigated this relationship in normal weight and overweight children, the last study [50] focussed on malnourished children, with results revealing that stunted/severely stunted boys were less sedentary on both school and non-school days.

## 4. Discussion

The purpose of this review was to provide an extensive search on PAP. Herein, our review encompassed (1) the putative definitions of PAP; (2) the instrumentation and techniques used to determine PAP; (3) the statistical procedures often used to analyse PAP; and (4) the associations among PAP and child health. Overall, the reviewed papers presented a moderate methodological quality (≥ 50%). Responses to Q3 were endorsed with the lowest score, meaning that none of the studies presented a clear definition regarding the meaning of PAP. However, the general mean score observed for Q4 (1.47) and Q7 (1.39) revealed that studies clearly presented the strategies used to measure PAP (notwithstanding, it was not clear what PAP means in the studies), as well as the conclusions synthetised the major results, with most of them indicating the implications/relevance of the studies, and suggesting directions for future work.

Notwithstanding the number of published papers concerning PAP, there is apparently no consensus regarding PAP’s real *substratum*. According to the Longman dictionary (p. 1120) [94], a pattern can be defined as “the regular way in which something happens, develops, or is done”. However, as outlined in this review, most papers did not intensively examine children’s PA at an intra-personal level, but typically focussed on describing/explaining/understanding their PA levels, i.e., their summaries, comparing groups and conditions, or even describing frequencies with which PA guidelines were achieved. In this context, a review by Welk et al. [95] presented children’s PAP according to its “highly transitory nature”. Here, the authors argued that PAP during childhood is better reflected by the accumulation of daily intermittent activity as opposed to being continuous in nature. The authors also reinforced that frequency, intensity, and duration are variables that are commonly used to characterise PAP. Welk et al. [95] also highlighted that a clear PAP definition is necessary, allowing researchers to investigate similar variables and, more importantly, avoiding the imprecise use of this term.

The instrumentation used to capture putative measurements of PAP varied substantially across studies, and all available “arsenal” was used. In fact, accelerometers, pedometers, heart rate monitors, questionnaires, and observational methods were employed. This means that, irrespective of the ways each study aims were formulated, there is no recognised agreement upon a standard instrument with which PAP could be best captured. As previously mentioned, it also reflects the absence of a unique and clear definition of PAP. For example, when PAP was investigated on a daily based routine, with the purpose to identify how PA changes between and/or within days, accelerometers or pedometers were the most used instruments. However, when researchers were interested in describing different types of activities children are usually engaged in, as well as the places where these took place, questionnaire or observational methods were used. As suggested by Welk et al. [95], given the unique aspects of children’s movement patterns, these patterns influence the existing measurement techniques to determine PA, leading to a wide range of instruments used to measure PA in children, all with different strengths and weakness [96].

Given that in the most of the reviewed papers, instruments were usually used to describe children’s PA levels (which has being used as a PAP definition), it would be expected that authors did not use specific statistical analyses procedures to determine PAP. In fact, few studies [14,23,40,71,74,92] mentioned the use of specific procedures to determine/analyse PAP. Yet, even in papers where specific procedures were mentioned to be used, it was possible to observe that the PAP definition was not always clear. For example, when profile analysis was used, the authors’ aim was to compare differences between groups [71,92], and using cluster analysis allowed them to group children according to their behaviour similarity [14]. On the other hand, using ratios provided information regarding the proportion of the time a child is engaged in more or less PA from different intensities in comparison with time in sedentary behaviours [40]. Despite the authors’ efforts to determine children’s PAP, most results do not reflect what PAP should mean, i.e., the pattern of a behaviour that ought to take into account the individual child streams of PA behaviours, rather than compare it with others or even to describe if, in the total amount of the time, the child is more or less active than sedentary.

The last aim of this review was to summarise the available research concerning the relationship between PAP and health markers. It is well-known that MVPA is positively associated within numerous physical and psychological health benefits [1]. Indeed, based on such evidence, recognised international guidelines suggest the minimum amount of time children and adolescents should be active on a daily basis to improve their health [2]. Most of the studies linking PAP and health were more interested in analysing the relationship between PA levels and health (or health indicators), but not PAP *per se*. PA markers such as MVPA, different intensities of PA children are engaged in, and the compliance with PA guidelines were used to associate PAP with health indicators. This suggests that information is still lacking on how PAP may influence the physical and psychological health and well-being of children. It is now evident that children who are more engaged in MVPA tend to be healthier, with a better metabolic profile than those who are less active [1]; yet, what it is not clear is how this relationship stands when considering different patterns. For example, is it best to: (1) be engaged in 60 min of continuous MVPA or in various time fractions alongside the day or (2) have a more erratic PAP profile (i.e., with random bursts of MVPA)? Such questions could probably be answered if a consensus existed on the exact meaning of PAP, which in all likelihood would guide research in a more enriching and impactful way.

The present review is not without limitations. First, we only considered children aged between 6 and 11 years; yet, including adolescent and/or adult data would probably be too extensive to synthesise in a single review. Second, the fact that we did not use other possible definition/terms during our search that may be used as a “physical activity pattern” synonym.

## 5. Conclusions

In conclusion, this systematic review shows that there is no consensus regarding a clear PAP definition whatever the instruments used to capture it. Furthermore, there apparently is no agreement on how best PAP should be analysed. Hence, there is an “urgency” for a formal and clear definition of PAP in order to guide researchers in future studies, highlighting the most useful instruments and statistical procedures to best capture these complex streams of behaviours. We then suggest that PAP can and should be used when aiming to probe similarities/dissimilarities and stabilities/changes in children PA at an intra-personal level, such that their streams of activity/sedentary behaviours may be searched for differences, or not, across a variety of conditions that apparently rule their lives, meaning that PAP should be used to best describe individual streams of personal behaviours, and not only PA intensities.

We suggest that physical activity research should focus on providing precise answers to putative links between different PAP and health risk profiles as well as health benefits. Furthermore, public health policies and guidelines should also consider these links rather than exclusively focus on average daily minutes. For example, how were these 60 min achieved? Do children maintain the same pattern of activities across the entire week, or do they vary in activities and intensities? Should they do intense or moderate activities during breaks, spread along the day (and if so, what is the duration of these breaks and in which periods of the day should they occur)? Yet, it is also important that school, families, and governments provide children opportunities to be safely active, during different moments of their days.

## Figures and Tables

**Table 1:** Quality criteria developed to analyse the papers.

	Question	Answer	Score
Q1	The aim(s) of the study(ies) is/are clearly set out	Yes = 2; Maybe = 1; No = 0	0–2
Q2	Characteristics of the participants are presented *(number of subjects, sex, age, country/city)*	Yes = 2; Maybe = 1; No = 0	0–2
Q3	Clear definition of physical activity pattern	Yes = 2; Maybe = 1; No = 0	0–2
Q4	Clear description of methods/strategy to determine physical activity pattern	Yes = 2; Maybe = 1; No = 0	0–2
Q5	Statistical procedures used to determine physical activity pattern *(not only to determine group difference)*	Yes = 2; Maybe = 1; No = 0	0–2
Q6	Results are detailed regarding to physical activity pattern *(not only present mean and standard deviation)*	Yes = 2; Maybe = 1; No = 0	0–2
Q7	Conclusions insightful *(clear, practical applications, and future directions)*	Yes = 2; Maybe = 1; No = 0	0–2
Total			0–14

**Table 2:** Summary of the studies that used accelerometers to assess PAP.

Continent	Country/Study	Sample	PAP Variables	Quality Score
Africa	South Africa (Walter, 2011 [49])	- 112 subjects - 8–12 years (mean age: 10.28 years)	- Time spent in SED, LPA, MPA, VPA - 30 min MVPA achievement	64.29%
Asia	Nepal (Williams et al., 2016 [50])	- 399 subjects - 6–18 years	- Time spent, and percentage of time, in SED, LPA, MPA and VPA (MVPA) - Achievement of daily MVPA (60 min/day) and mean hour spent in MVPA - School days and non-school days (Saturday)	64.29%
	China (Huang et al., 2019 [55])	- 599 subjects - Mean age: 8.5–8.9 years	- Time spent in MVPA and sedentariness - MVPA on weekdays, weekend days, and mean week	57.14%
Europe	Portugal (Guerra et al., 2003 [36])	- 157 subjects - 8–16 years (55 aged 8–10 years)	- Average daily time in MVPA - Percentage of MVPA achieved	57.14%
	Netherlands (Hoos et al., 2004 [37])	- 20 subjects - Mean age: 8.6 years	- Time spent in LPA, MPA, and high-intensity activity	50%
	UK (Page et al., 2005 [45])	- 133 subjects - 9–11 years	- Minutes/hour in at least MPA - Hourly activity - Schooldays and weekend days	71.43%
	France (Baquet et al., 2007 [31])	- 34 subjects - 8–10 years	- Time in LPA, MPA, VPA, and VHPA - Daily numbers of physical activity bouts of different duration, for each intensity levels	64.29%
	Sweden (Nyberg et al., 2009 [44])	- 1293 subjects - 6–10 years	- Overall mean physical activity - Tertiles (sex specific) based on mean total activity counts: low, medium, high - Week and weekend days - Segments of the day	71.43%
	UK (Ridger et al., 2010 [47])	- 110 subjects - 9–10 years	- Hourly amount of time/day spent equal to or above 4 km/h (PA_≥4_) - Week and weekend days	71.43%
	Spain (Aznar et al., 2011 [30])	- 221 subjects - 136 aged 9 years; 85 aged 15 years	- Minutes in SED, LPA, MPA, and VPA - Weekday and weekend day - MVPA achievement	64.29%
	France (Blaes et al., 2011 [33])	- 361 subjects - Mean ages: 4.3-12.6 years (preschoolers, primary and junior)	- Time in LPA, MPA, VPA, and VHPA, for each of the seven days - Percentage of children achieved the MVPA guidelines (60 min/day, five days a week)	57.14%
	UK (Jago et al., 2011 [38])	- 472 subjects - 10–11 years	- Mean counts/minute - Mean time in MVPA	50%
	UK (Fairclough et al., 2012 [35])	- 223 subjects - 10–11 years (mean age: 10.7 years)	- Mean minutes of MPA and VPA, at different day segments - Time in MVPA on each of the five days (achievement of 60 min/day) - LOW: achieve the recommendation < 50% of their valid days; HIGH: achieve the recommendation for ≥ 50% of their valid days	64.29%
	Spain (Laguna et al., 2013 [12])	- 761 subjects (487 children; 274 adolescents) - 9 years and 15 years	- Time in SED, LPA, MPA, VPA, and VHPA - Whole week, weekdays, weekend days - Number of days 60 min MVPA achieved	71.43%
	Finland (Arto, 2015 [29])	- 76 subjects - 10–13 years (mean age: 11.43)	- School day and out-of-school - Total LPA and LPA in different day segments	71.43%
	Estonia (Mooses et al., 2016 [42])	- 472 subjects - 6–13 years - First (7–8 years) and secondary (10–13 years) children	- Time in MVPA: whole day and for different day segments - Average method: considered compliant with achieve MVPA guidelines considering the all measured days mean - Days method: considered compliant when MVPA guidelines achieved for at least 4 measured days	71.43%
	Italy (Pau et al., 2017 [46])	- 169 subjects - Mean age: 8.6 years	- Time in SED, LPA, MPA, VPA (MVPA) - Percentage of time spent in each physical activity category and number of steps, for each day segment	57.14%
	Norway (Aadland et al., 2018 [51])	- 841 subjects - Mean age: 10.2 years	- Mean time in SED, LPA, and VPA - 15 different bout durations between 1 s and ≥60 min	64.29%
	UK (McLellan et al., 2019 [56])	- 188 subjects - 7–12 years	- Mean time in SED, LPA; MPA, VPA (MVPA) - Weekdays (before school, during school, after school) and weekend days (morning and after-evening)	71.43%
North American	Mexico (Jáuregui et al., 2011 [39])	- 217 subjects - 5–6 years at baseline (mean ages: boys, 5.9 years; girls, 6 years)	- Minutes in MVPA - Week and weekend days - At school and off-school hours	71.43%
	Canada (Stone et al., 2012 [48])	- 713 subjects - Mean age: 11.1 years	- Total physical activity (counts/min), mean counts, time spent in SED (percentage of the day) and minutes in LPA and MVPA - School days and weekend	50%
	USA (Miller et al., 2013 [28])	- 193 subjects - Mean age: 8.8 years	- Counts per segment of day	71.43%
	USA (Butte et al., 2014 [34])	- 282 subjects - 8–10 years	- Time in SED, LPA, MPA, and VPA - Percentage of time in SED and MVPA	57.14%
	USA (Loprinzi et al., 2015 [40])	- 2644 subjects - Children: ages 6–11 years - Adolescents: ages 12–17 years	- Total time in physical activity - Time in sedentary, LPA, MPA, VPA (MVPA) - Achieve MVPA guidelines - Movement patterns: (1) meeting MVPA guidelines and engaging in relatively more LPA than SB (2) meeting MVPA guidelines and engaging in relatively less LPA than SED (3) not meeting MVPA guidelines, but engaging in relatively more LPA than SED (4) not meeting MVPA guidelines and engaging in relatively less LPA than SED	71.43%
	USA (Fetter et al., 2018 [52])	- 92 subjects - 9–10 years	- Mean time spent in SED, easy PA; MPA, VPA (MVPA), and VVPA - Fall and Spring	64.29%
	USA (Saint-Maurice et al., 2018 [53])	- 291 subjects - Children: mean ages 9.7 (elementary-school) and 11.7 (middle-school) - Adolescents: mean age 15.7 years	- Mean minutes and percentage time in MVPA - Segments of the day, school time (transportation to school, recess, PE, lunch, transportation from school), out-of-school (before school, after school, evening, weekend)	71.43%
South America	Brazil (Bielemann et al., 2013 [32])	- 239 subjects - 4–11 years	- Intensity and prevalence of physical activity - Mean daily minutes in SED, LPA, MPA, and VPA - Insufficient physical activity (yes/no): 60 min/day MVPA - Mean daily time - Mean counts/minute	57.14%
	Colombia (Briceño et al., 2019 [54])	- 19 subjects - 6–8 years (followed for 2 years)	- Average of active energy expenditure - Min/day in rest, light, moderate, or vigorous active	50%
Oceania	New Zealand (McGall et al., 2011 [41] )	- 60 subjects - Mean age: 8.3 years	- Total daily activity, time in SED, LPA, MPA, and VPA - Days of MVPA guideline achieved, and total MVPA - Segments of the day, school time, week and weekend days - Mean weekdays, Saturday, Sunday	64.29%
	Australia (Ridger et al., 2015 [9])	- 326 subjects - 8–11 years	- Time in MVPA - Frequency and duration of time accumulated in bouts (≥ 1 min) of MPA and VPA	64.29%
	Australia (Remmers et al., 2017 [8])	- 307 subjects - 8–11 years	- LPA, MPA, and VPA - Daily total minute in MPA and VPA - Day-to-day variation (intra-individual variation)	78.57%
Multi-Country	Kenya and Canada (Muthuri et al., 2014 [43])	- 1096 subjects (555 Kenyan and 541 Canadian) - 9–11 years	- Time spent in sedentary, light, and MVPA - By time of the day - Week and weekend days	71.43%

LPA, light physical activity; MPA, moderate physical activity; MVPA, moderate to vigorous physical activity; SED, sedentariness; SES, socioeconomic status; VHPA, very high physical activity; VPA, vigorous physical activity; VVPA, very vigorous physical activity.

**Table 3:** Summary of the studies that used pedometers to assess PAP.

Continent	Country/Study	Sample	PAP Variables	Quality Score
Europe	Poland (Czajka et al., 2015 [61])	- 221 subjects (both sexes) - 6–7 years	- Average total daily steps at the week - Average total daily steps on weekdays - Average total daily steps on weekend days - Daily steps - Achievement of recommended steps/day	71.43%
North America	USA (Johson et al., 2007 [22])	- 176 subjects (both sexes) - 8–12 years (mean age: 9.8 years)	- Mean steps at school day - Mean activity time (min) at school day	50.0%
	USA (Dauenhauer et al., 2011 [62])	- 71 subjects (both sexes) - 8–11 years	- Mean full day steps: (1) average total daily steps (2) average total daily steps on weekdays (3) average total daily steps on weekend days (4) average total daily steps on 0 min physical education days (5) average total daily steps on 30 min physical education days (6) average total daily steps on 60 min physical education days - Partial-day steps: (1) average 30 min physical education class steps (2) average 60 min physical education class steps (3) average steps on 30 min physical education days, excluding steps in physical education (4) average steps on 60 min physical education days, excluding steps in physical education	64.29%
	USA (Brusseau et al., 2011 [57])	- 829 subjects (both sexes) - Mean age: 9.6 years	- Average total daily steps - Average steps at school - Average steps at physical education - Average steps at recess time - Average steps at lunchtime - Average steps outside schools	64.29%
	USA (Brusseau et al., 2011 [60])	- 363 subjects (both sexes) - 8–11 years	- Average total daily steps (all days) - Average steps at weekend days - Average steps on physical education days - Average steps on non-physical education days	64.29%
	USA (Brusseau et al., 2013 [59])	- 77 subjects (both sexes) - Mean age: 11.26 years	- Average total daily steps at weekdays - Average total daily steps at weekend days - Average steps at school - Average steps at physical education - Average steps at lunchtime - Average steps outside schools	71.43%
	USA (Brusseau, 2015 [58])	- 287 subjects (both sexes) - Mean age: 9.48 years	Data collected at two seasons (Fall and Spring) - Average total week daily steps - Average total daily steps on weekdays - Average total daily steps on weekend days - Average total daily steps on physical education days - Average steps on physical education - Average steps at lunchtime - Average steps at recess time - Average steps in school - Average steps out of school - Average total daily steps on weekdays - Average total daily steps on weekend days	71.43%

**Table 4:** Summary of the studies that used heart rate monitors to assess PAP.

Continent	Country/Study	Sample	PAP Variables	Quality Score
Africa	Senegal (Bénefice, 1992 [66])	- 100 subjects (both sexes) - 10–14 years (41 subjects aged 10–11.9 years)	- Frequency, intensity, and duration of physical activity - < 125 bpm: unstressful activity - ≥ 125 bpm and ≤140 bpm: moderate activity (60% of maximum HR) - > 140 bpm: vigorous (70% of maximum HR)	64.29%
	Senegal (Bénéfice and Ndiaye, 2005 [67])	- 99 children (both sexes) - 10–13 years (mean age: 11.1 years) - 43 adolescents girls - mean age: 15.5 years - 30 women - 17–40 years	- % of time spent below the resting value of the exercise test - % of time spent between the resting value and the flex-HR - % of time spent between the flex-HR and the maximal HR of the exercise test - % of time spent above the maximal HR - % of time spent under or over the flex-HR *flex-HR: the mid-value between the lowest HR value in a sitting position (generally during the second minute) and the lowest HR value of the first minute of exercise	64.29%
	Tunisia (Zarrouk et al., 2009 [70])	- 90 subjects (both sexes) - 8–11 years	- Average daily level of physical activity (TEE/RHR) - SED (< 30% HRR) - MPA (30–50% HRR) - VPA (50–70% HRR) - High intensity (>70% HRR)	64.29%
Europe	UK (Armstrong and Bray, 1990 [63])	- 24 subjects (both sexes) - 10–11 years	- Frequency, intensity, and duration of physical activity - Periods of 5, 10, and 20 min in > 139 bpm and >159 bpm - Two seasons: Autumn and Summer	50%
	UK (Armstrong and Bray, 1991 [64])	- 132 subjects (both sexes) - mean age: 10.7 years	- Frequency, intensity, and duration of physical activity - Periods of 5, 10, and 20 min in > 139 bpm and > 159 bpm	57.14%
	UK (Armstrong et al., 1996 [65])	- 129 (from 745) subjects - Mean ages: 11.1 years (boys) and 10.9 years (girls)	- Frequency, intensity and duration of physical activity - Periods of 5, 10, and 20 min in > 139 bpm and > 159 bpm	57.14%
	Belgium (Massin et al., 2004 [69])	- 200 subjects (both sexes) - 3–16 years - Preschool (59 subjects): 3–6 years - Schoolchildren (105 subjects): 7–12 years - Teenagers (36 subjects): 13–16 years	- Frequency, intensity, and duration of physical activity - LPA: 20%–40% HRR - MPA: 40%–50% HRR - High intensity activity: >50% HRR	64.29%
North America	USA (Gilliam et al., 1982 [71])	- 59 subjects (both sexes) - 6–7 years	- HR for each 5 min interval - Time in HR categories: (1) LPA: 80–109 bpm (2) MPA: 110–159 bpm (3) High: ≥ 160 bpm - Energy expenditure (estimated)	78.57%
	USA (Loftin et al., 1998 [68])	- 16 girls (from 32) - Mean age: 9.8 years	- Minutes over 2 days of MVPA (≥ 60% MHRR) and VPA (≥ 75% MHRR) - Sustained periods of 5, 10, and 20 min in MVPA and VPA	57.14%

bpm, beats per minute; FFM, fat free mass; FM, fat mass; HR, heart rate; HRR, heart rate reserve; LPA, light physical activity; MPA, moderate physical activity; MVPA, moderate to vigorous physical activity; PAI, physical activity intensity; RHR, resting heart rate; SED, sedentariness; TEE, total daily energy expenditure VPA, vigorous physical activity.

**Table 5:** Summary of the studies that used questionnaires and observation to assess PAP.

Continent	Country/Study	Sample	PAP Variables	Quality Score
Africa	Nigeria (Nwogu et al., 2019 [83])	- 457 subjects - 6–14 years (mean ages: 11.34–11.53)	- Physical activity	57.14%
Asia	Singapore (Schmidt et al., 1998 [78])	- 1579 subjects (both sexes) - 6–17 years	- Days in hard and easy exercise - Screen daily hours - Sports involvement (annual) - Five groups classification: inactive; relatively no activity; light activity; moderate activity; vigorous activity	57.14%
	Thailand (Amini et al., 2009 [73])	- 85 subjects (both sexes) - 10–12 years	- Recall of different activities performed in a typical day, from morning until going to bed - Activities classified as light, moderate, or heavy	57.14%
	India (Swaminathan et al., 2011 [77])	- 256 subjects (both sexes) - 8–15 years (two age groups: ≤ 11 years, > 11 years)	- Time spent and the daily, weekly, or monthly frequency of each activity - SED and MVPA - Achievement of MVPA 60 min/day - Total energy expenditure (MET/min) - Week and weekend days - Sports involvement - Transportation mode	57.14%
	India (Esht et al., 2018 [81])	- 234 subjects - 8–14 years (76 aged 8–10 years)	- Physical and sedentary activities: physical activity during school, involvement in games (in and out school hours), travel to/from school, household activities, TV/video viewing, computer games, tuitions, homework, sleep time - Physical activity level (based on MET’s) categories: nil/fairly LPA; sedentary/LPA; MPA; VPA/HPA - MVPA achievement	57.14%
Europe	UK (Dickenson, 1986 [79])	- 500 subjects (both sexes) - 11–16 years	- Frequency, duration and intensity of VPA - Children classified as “not active”, < 5 min VPA, < 30 min VPA	42.86%
	UK (Riddoch et al., 1991 [80])	-3211 subjects (both sexes) - 11–18 years	- Frequency, duration, and intensity of “light-to-moderate” and VPA or exercise	50%
	Cyprus (Bathrellou et al., 2007 [75])	- 1140 subjects (both sexes) - 10–12 years (mean age: 10.67 years)	- Frequency and duration of physical and sedentary activities on week and weekend days - At school and out of school - Time spent in MVPA - Physical activity lifestyle and behaviours	57.14%
	Greece (Antonogeorgos et al., 2010 [74])	- 700 subjects (both sexes) - 10–12 years	- Frequency and duration of physical activities in a typical week (mainly for the past 7 days) - Free time activities - Sedentary activities - Sports participation - Type of activity, time spent, intensity, and weekly frequency - Daily physical activity evaluation (week and weekend days) - Pattern of physical activity, compared to the last week (daily): “increasing” or “decreasing” - Non-active (≤ 37 kcal × kg^−1^×day^−1^) and active (> 37 kcal × kg^−1^ × day^−1^)	85.71%
	Spain (Muntaner-Mas et al., 2017 [11])	- 3164 subjects (both sexes), from which 691 children - 10–16 years (children: 10–11 years)	- Weekly hours of physical activity practice - Places to practise physical activity - How to practise physical activity - Reasons to enrol in physical activity for the first time	57.14%
	UK (Bosch et al., 2019 [82])	- 2171 subjects (both sexes) - 5–11 years	- Frequency of children participation in extracurricular PA (in a whole week) - Transport when commuting from home to school (active or passive or mixed commuting)	64.29%
North America	USA (Corbin and Pletcher, 1968 [84])	- 50 subjects (both sexes) - Mean ages: 9.7-10 years	- Physical education period: unorganised, low organised, and organised play - Mean physical activity index - Mean scores of percent of time in activity	50%
	USA (Berman et al., 1998 [23])	- 15 subjects (both sexes) - 6–10 years (mean age: 8.3 years)	- Low, medium, and high intensity activities (estimated VO2(ml/min/kg) ) - Number of bouts in each activity intensity - Average bout duration - Estimated energy expenditure as O_2_ uptake	78.57%
	USA (Ha et al., 2005 [72])	- 1018 subjects (both sexes) - 10–12 years (mean age: 10 years)	- Daily hours/minutes in physical activities and watching TV - Physical activity guidelines achievement (60 min/day)	57.14%
	Mexico (Colín-Ramírez et at., 2010 [76])	- 498 subjects (IG: 245; CG: 253) (both sexes) - 8–10 years	- Days involved in MVPA and MPA, in last week - Hours spent in sedentary activities on the previous day	57.14%
	Mexico (Vergara-Castañeda et al., 2010 [10])	- 83 subjects (and one of the parents) (both sexes) - Mean age: 9.4 years	- LPA and MPA - Leisure activities (screen activities)	50%
	USA (Brewer and Kimbro, 2014 [27])	- 17,500 subjects (both sexes) - Mean ages: 73.4- 74.9 months	- Days/week (0–7) of VPA	57.14%

CG, control group; IG, intervention group; HPA, heavy physical activity; LPA, light physical activity; MPA, moderate physical activity; MVPA, moderate to vigorous physical activity; SED, sedentariness; VPA, vigorous physical activity.

**Table 6:** Summary of the studies that used combined instruments to assess PAP.

Continent	Country/Study	Sample	PAP Variables	Quality Score
Asia	India (Krishnaveni et al., 2009 [87])	- 103 subjects (both sexes) - Mean age: 6.6 years	- Time spent in SED, LPA, MPA, and VPA	71.43%
	China/Hong Kong (Gao et al., 2015 [90])	- 68 subjects (both sexes) - 10–11 years	- Total daily steps (sum of all segments) - Total daily steps without PE class (school days without PE classes) - Total daily steps with PE class (school days with PE classes) - Step counts in school - Step counts out of school - Step counts during recess - High and low-active groups (according to daily step median values cut-points) - Travel mode	71.43%
	Turkey (Okur et al., 2019 [88])	- 40 subjects - 6–18 years (mean ages: 10.7-10.75)	- Total energy expenditure - Mean physical activity energy expenditure	57.14%
Europe	UK (Cooper et al., 2003 [85])	- 141 subjects (both sexes) - Mean age: 10.4 years	- Time spent in MVPA - MVPA hourly (7:00–21:00) - Counts/min - MVPA in different segments of the day - Weekdays and weekend days - Travel mode	64.29%
	Denmark (Cooper et al., 2005 [86])	- 323 subjects (both sexes) - Mean age: 9.7y	- Time spent in MVPA - MVPA hourly (7:00–21:00) - MVPA in different segments of the day - Weekdays and weekend days - Travel mode	71.43%
	UK (Al-Nakeeb et al., 2007 [93])	- 47 subjects (both sexes) - 9–10 years	- Frequency, intensity, and duration of physical activity - HR minute by minute - week and weekend days - MVPA > 139 bpm: average time and percentage of the total recorded period of time - Periods of 5 min, 10 min, and 15 min	71.43%
	UK (Jago et al., 2010 [14])	- 761 subjects (both sexes) - 10–11 years	- Counts/minute - Time in sedentary and MVPA - Week and weekend days	78.57%
	Czech Republic (Sigmund et al., 2014 [89])	- 338 subjects (both sexes) - 9–11 years	- Step count - Minutes in MVPA - MVPA HR response (> 60% of MHR) (minutes)	64.29%
	Switzerland (Bürgi et al., 2016 [6])	- 83 subjects (both sexes) - Mean age: 8.5 years	- Time in SED, MPA, and VPA - % of time in MVPA and SED - Activity in different settings: outside (outside the urban area); home; school own; school other; park; sport; street; other	71.43%
North America	USA (Gilliam et al., 1981 [92])	- 40 subjects (both sexes) - 6–7 years	- HR for each 5 min interval - Time in HR categories: (1) ≤ 80 bpm (2) 80–100 bpm (3) 101–120 bpm (4) 121–140 bpm (5) 141–160 bpm (6) 161–180 bpm (7) > 180 bpm	71.43%
	USA (Vadivelo et al., 2009 [91])	- 35 subjects (both sexes) - 8–10 years	- Time in physical activity and sedentary activities - Comply steps/day recommendation - Steps per season	50%

bpm, beats per minute; HR, heart rate; LPA, light physical activity; MHR, maximum heart rate; MPA, moderate physical activity; MVPA, moderate to vigorous physical activity; PE, physical education; SED, sedentariness; SES, socioeconomic status; VPA, vigorous physical activity.

## Supplementary Materials

The following are available online at https://www.mdpi.com/1660-4601/17/16/5837/s1, Figure S1: Network interactions among authors from the revised papers.
