# Supplementary figures and images for: A Systematic Review of Children’s Physical Activity Patterns: Concept, Operational Definitions, Instruments, Statistical Analyses, and Health Implications
Source: Int J Environ Res Public Health. 2020 Aug 12;17(16):5837. doi: 10.3390/ijerph17165837 (PMC7459930; doi:10.3390/ijerph17165837)

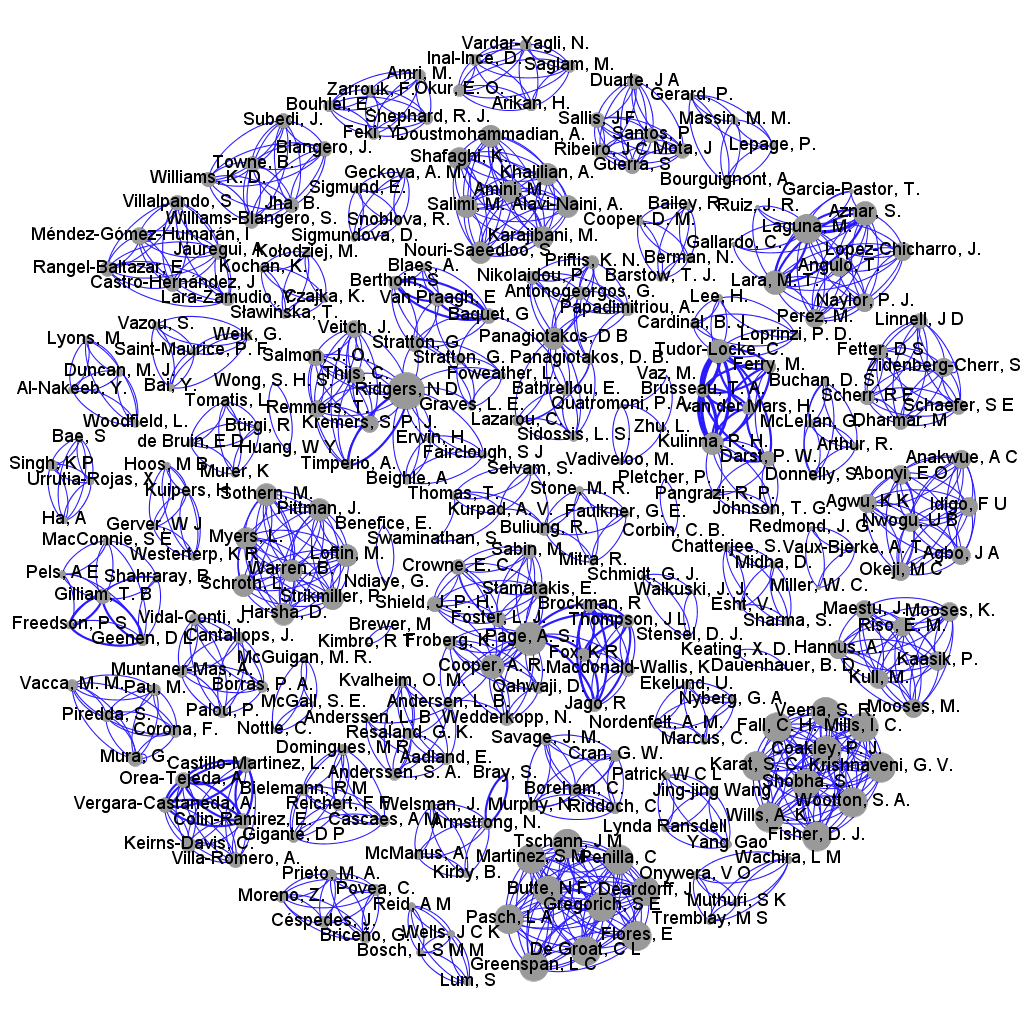

Supplement: Supplementary file 1 [file ijerph-17-05837-s001.zip › ijerph-853898-supplementary.png]
